# A Novel ENU-Induced Mutation in Myo6 Causes Vestibular Dysfunction and Deafness

**DOI:** 10.1371/journal.pone.0154984

**Published:** 2016-05-12

**Authors:** Elaine Y. M. Wong, Chelsea Y. Xu, Manisha Brahmachary, Pin-Xian Xu

**Affiliations:** 1 Department of Genetics and Genomic Sciences, Icahn School of Medicine at Mount Sinai, New York, New York, United States of America; 2 Developmental and Regenerative Biology, Icahn School of Medicine at Mount Sinai, New York, New York, United States of America; University of South Florida, UNITED STATES

## Abstract

Mouse N-ethyl-N-nitrosourea (ENU) mutagenesis has generated many useful animal models for human diseases. Here we describe the identification of a novel ENU-induced mouse mutant strain *Turner* (*Tur*) that displays circling and headtossing behavior and progressive hearing loss. *Tur/Tur* homozygous animals lack Preyer and righting reflexes and display severe headtossing and reaching response defect. We mapped the *Tur* mutation to a critical region of 11 cM on chromosome 9 that includes myosin VI. Direct sequence analysis revealed a c.820A>T substitution in exon 8 of the *Myo6* gene that changes amino acid Asn200 to Ile (p.N200I) in the motor domain. Analysis of inner ear hair cells by immunohistochemistry, scanning electron microscopy and histology revealed degeneration of hair cells in the inner ear and structural malformation of the stereocilia in the cochlea of *Turner* homozygous mutant mice. Our data indicate that this novel mouse strain provides a useful model for future studies on the function of myosin VI in mammalian auditory and non-auditory systems and in human syndromes.

## Introduction

Deafness is the most common sensory disorder in humans, produced primarily by damage to the inner ear sensory hair cells and their associated spiral ganglion neurons. Over the past several decades, large numbers of new mouse mutants with deafness or vestibular dysfunction have been generated through large-scale ENU and other mutagenesis programs. This has led to discovery of novel candidate genes for human deafness and more animal models for studying human syndromes.

Myosin VI belongs to a group of proteins called unconventional myosins, which are molecular motors that interact with actin to function as either actin-based anchors or transporters [1], and is essential for hearing in both humans and mice. The *Myo6* gene was first reported to be mutated in *Snell’s waltzer* mice that exhibited circling and headtossing behavior and deafness due to loss of hair cells [2]. It encodes a 1265 amino acid protein (140 kD) that consists of an N-terminal motor domain involved in actin-binding and movement, a calmodulin interacting neck domain and a C-terminal tail domain that often connects to various cargo associated proteins [3]. Myosin VI is expressed in the inner ear hair cells and its expression in the hair cells has been found to be localized to the base of the stereocilia in the cuticular plate, leading to the proposed role of myosin VI as responsible for anchoring the stereocilia to the cuticular plate [4]. Mutations in the human *MYO6* gene are associated with a dominant nonsyndromic deafness called DFNA22 [5] and a recessive form of hearing loss called DFNB37 [6, 7]. Mutations that cause single amino acid changes or truncation of the myosin VI protein were identified from patients or mouse models and these mutations are likely to alter the function of myosin VI, which leads to disruption of the structure and organization of stereocilia and hearing loss. Although myosin VI is essential for normal inner ear function, its exact role is still not fully understood.

Here we report *Turner* (*Tur*), a novel ENU-induced dominant point mutation c.820A>T in exon 8 of the *Myo6* gene that changes amino acid Asn200 to Ile (p.N200I) in the motor domain, causing headtossing and circling behavior and hearing impairment.

## Materials and Methods

### Mice and behavioral analysis

The founder mouse carrying the *Tur* mutation was generated in a large-scale ENU mutagenesis program at the McLaughlin Research Institute [8]. Male C57BL/6J mice were injected with three doses of 80 mg/kg ENU at weekly intervals, allowed to recover and outcrossed to C3HeB/FeJ females. The male *Turner* founder of F1 offspring was discovered because of its headtossing and circling behavior.

A total of 72 mice were used for behavioral tests: 24 controls (12 males and 12 females), 24 *Tur/+* (12 males and 12 females) and 24 *Tur/Tur* (12 males and 12 females). A custom built click box was held above the mouse to deliver a calibrated 20 kHz tone burst at an intensity of 90 dB sound pressure level (SPL) and the presence of an ear flick response (Preyer reflex) was recorded. The click box test can only identify mice that have a severe or profound hearing impairment, and not those with mild to moderate deafness. Other behavioral tests including reaching response, contact righting, and swimming tests were also performed [9].

All procedures involving animals were approved by Animal Care and Use Committee at the Mount Sinai School of Medicine (#06–0807). All mice used for this study were monitored daily and no animals became ill or died prior to the experimental endpoint or received medical treatment.

### Auditory-evoked brainstem response (ABR) testing

We used a computer-assisted evoked potential system to obtain ABR thresholds for tone pips at 5, 8, 11, 16, 22, 32 and 45 kHz (tone pip duration 5 ms; repetition rate 30/s) and averaged responses to 512 pips of alternating polarity as described before [10].

### Genetic mapping and sequence analysis

*Tur/+* F1 offspring founder on a mixed C57BL/6J and C3HeB/FeJ genetic background was backcrossed to C3HeB/FeJ mice. N2 offspring were identified as mutant if they displayed a strong phenotype that consisted of headtossing, hyperactivity, circling behavior and severely compromised performance in a swimming and reaching response test. Genomic DNA were isolated from 11 affected and 5 unaffected G3 offspring as well as from the N2 breeding pair and analyzed with SNP genotyping (768 SNP panel) at Brigham and Women’s Hospital, Harvard Medical School in Boston.

After linkage to chromosome 9 between rs6292345 (75.3386 Mb) and rs3698443 (87.1572 Mb) was discovered, whole-exome sequencing of DNA isolated from wild-type C3HeB/FeJ, wild-type C57BL/6J, *Tur/+* and *Tur/Tur* homozygous G3 mice (3 mice for each genotype) was performed at Otogenetics (www.otogenetics.com). Bioinformatic analysis of sequencing data was focused on the mutation region on Chromosome 9.

After a mutation was identified in the exon 8 of the *Myo6* gene by whole-exome sequencing, two primers located in intron 7 and 8 respectively of the *Myo6* gene (intron 7 forward: 5’AGAGATGTAATATGTATATTG and intron 8 reverse 5’CATACAAACATACAATAATAG) were synthesized and PCR was performed to amplify the exon 8 of the *Myo6* gene. PCR products were purified and directly sequenced to confirm the mutation.

### Inner ear morphological analysis

We anaesthetized mice with urethane and perfused with 10% formalin, filled auditory bullae with 10% formalin, 1% acetic acid and immersed the entire head in this fixative for 3 days. We decalcified specimens in formic acid (8 N), dehydrated, embedded, sectioned in paraffin (8 μm) and stained with haematoxylin and eosin as described [11]. We examined five mutants and three wild-type controls.

### Immunofluorescence and phalloidin staining

Immunofluorescence of whole-mount cochleae harvested from two wild-type and three *Tur/Tur* at P0, P14 and P21 was performed as described [12]. Rhodamine-conjugated phalloidin (Invitrogen-Molecular Probes), rabbit anti-myosin VIIa (Proteus Biosciences) and rabbit anti-myosin VI (Proteus Biosciences Inc) with anti-rabbit secondary antibody conjugated to Cy3 were used in this study.

### Scanning electron microscopy (SEM)

Three wild-type controls and three homozygotes at P0 and three wild-type, two heterozygotes and three homozygotes at P21 were investigated by SEM. Freshly isolated ears were locally perfused through oval and round windows with 2.5% glutaraldehyde in 0.07 M sodium cacodylate buffer pH 7.4 with 3 mM CaCl_2_ and then fixed for 4 to 5 hours rotating at 4°C in the same fixative. Ears were washed in 0.1 M phosphate buffer and, for preparation of the organ of Corti, the outer bony shell and stria vascularis were removed. Samples were prepared using the osmium tetroxide-thiocarbohydrazide method [13]. Processed cochleae were then dehydrated by critical point drying using pressurized carbon dioxide [14]. Dried samples were mounted onto 0.5-inch aluminum stubs using silver DAG electro-conductive paint (Agar Scientific, Stansted, Essex, England), sputter-coated with gold, and examined under a Phillips XL30 scanning electron microscope at 5 kV. Adobe Photoshop was used for analyzing post-acquisition image.

## Results

### Turner is a dominant mutation causing vestibular dysfunction and hearing loss

The *Turner* founder was initially discovered in a large-scale screen of ENU-mutagenized mouse strains because of its mild headtossing and circling behavior, which was shown to have a dominant inheritance by outcrossing the founder C57BL/6J-*Tur/+* to wild-type C3HeB/FeJ mice. *Tur/+* mice at ~7–8 weeks of age also failed the swimming test and showed a weaker response to Preyer reflex test (Table 1).

**Table 1:** The penetrant rate for gross and behavioral abnormalities of *Turner* mice.

Phenotype	# of animals* (total 33 mice from *Tur*/+ x *Tur*/+ matings		
	8 (24.4%)	17 (51.5%)	8 (24.4%)
Abnormal Preyer reflex response:			
7 weeks old	0	12 (weaker, 70.6%)	8 (no response, 100%)
Reduced pinna size:			
Bilateral reduction	0	0	0
Unilateral reduction	0	0	0
Circling response:			
5 weeks old	0	2 (11.8%)	8 (100%)
7 weeks old	0	17 (100%)	8 (100%)
Headtossing:			
5 weeks old	0	0	8 (100%)
7 weeks old	0	12 (mild, 70.6%)	8 (severe, 100%)
Abnormal reaching response:			
7 weeks old		12 (70.6%)	8 (100%)

* Animals that exhibited headtossing also failed in reaching response, exhibited circling behavior and have an abnormal Preyer reflex.

Behavioral tests of the offspring of *Tur/+* × *Tur/+* matings at adult stage revealed that while approximately half of the offspring (17/33) exhibited behavioral abnormalities similar to those observed in the *Tur/+* heterozygous mice (Table 1), 8 out of 33 offspring (~24.4%) showed more severe headtossing and circling behavior and neither a contact righting response nor Preyer reflex (Table 1), indicating severe vestibular dysfunction and hearing impairment. These phenotypic observations together with Mendelian inheritance indicate that these severely affected mice were likely homozygotes.

**Fig 1:**
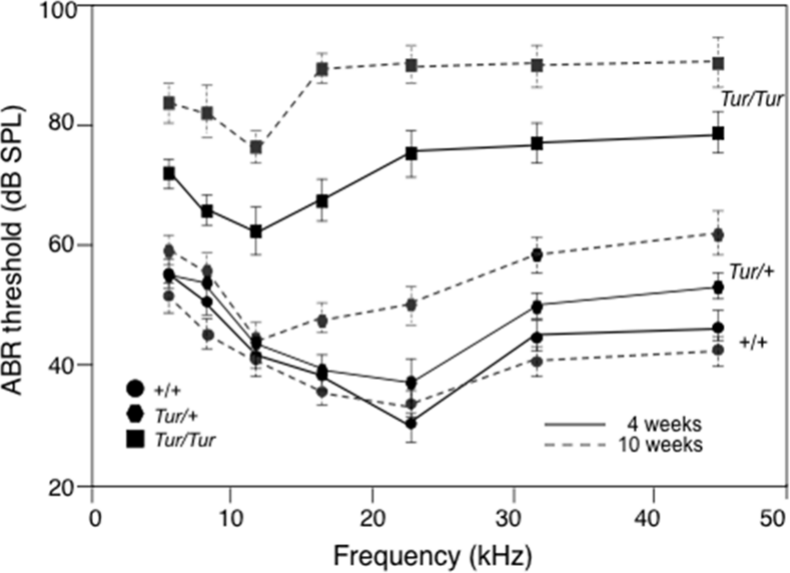
ABR threshold measurements of mice at 4 (unbroken lines) and 10 (broken lines) weeks of age. (A) Average threshold ± s.e.m. for wild-type (+/+) at 4 weeks (n = 3) and 10 weeks (n = 6), *Tur* heterozygous *(Tur/+)* at 10 weeks (n = 8) and homozygous ears (*Tur/Tur*) at 4 weeks (n = 5) and 10 weeks (n = 6) of age. Averages of 90 dB SPL were used for thresholds beyond the upper limit of the sound system. The *Tur/+* mice at 10 weeks of age had mild hearing loss in both ears (threshold shifted by 10–20 dB between 16 and 45 kHz), while *Tur/+* mice at 4 weeks of age (n = 5) had relatively normal hearing. However, all homozygous mice at 4 weeks of age had severe hearing loss (threshold shift by ~20–40 dB), while homozygous mice at 10 weeks of age had >35 dB loss between 5–11 kHz and >50 dB loss between 16–45 kHz.

Auditory-evoked brainstem response (ABR) thresholds confirmed that homozygous mice (n = 6) had profound hearing loss at 10 weeks of age (Fig 1), while heterozygous mice (n = 8) showed moderate hearing impairment with an ABR threshold shift to ~5 dB (at lower frequency) and 20 dB (at higher frequency) higher than that of normal mice (n = 6) (Fig 1). However, at 4 weeks of age, heterozygotes (n = 5) did not exhibit obvious hearing loss or had very mild hearing loss with ABR threshold shift to ~5 dB higher than that of control mice at high frequency (Fig 1). Of five 4-week homozygous mice (n = 5) analyzed, all had severe hearing loss in both ears with an ABR threshold shift to ~22 dB (at lower frequencies) to 40 dB (at higher frequencies) than that of normal mice (n = 3) (Fig 1). Thus, the *Turner* mutation causes progressive hearing loss in mice.

### Turner mutant mice have a point mutation in the *Myo6* gene on chromosome 9

**Fig 2:**
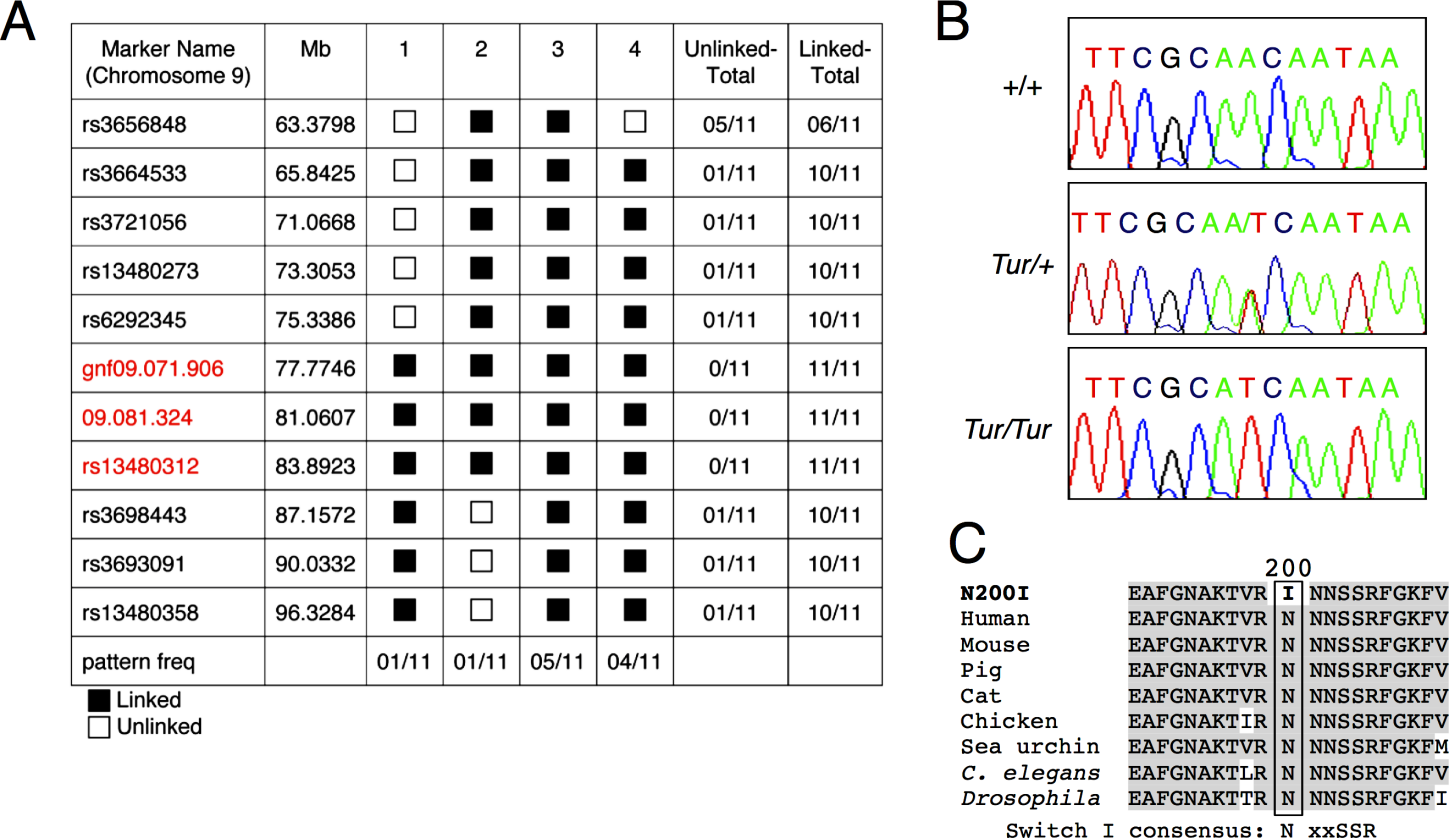
The *Turner* mutation in myosin VI. (A) Pattern program output of the results of 11 markers located between 63.3798 Mb and 96.3284 Mb, tested on DNA derived from 11 affected (9 were G3 offspring and 2 were the N2 breeding pair), that showed recombination between three markers (gnf09.071.906, 09.081.324 and rs13480312). The *Tur* mutation was determined to lie between marker rs6292345 (75.3386 Mb) and rs3698443 (87.1572 Mb). The black box (linked) indicates mice heterozygous for the C3H and the C57; the white box (unlinked) indicates mice homozygous for the C3H (patterns 1 and 2) or the C57 (pattern 4). (B) Sequence analysis of the *Turner* genomic DNA revealing a 820A>T transversion in exon 8 (NM_001039546). Control DNA shows a single A peak and heterozygous DNA (*Tur/+*) shows both A and T peaks, but homozygous DNA (*Tur/Tur*) shows a single T peak. (C) The p. N200I single amino acid substitution. Alignment of a portion of myosin VI protein from various species revealing conservation of the N200 residue. The switch I loop consensus sequence motif within the motor domain is indicated.

Linkage for the *Tur* mutation was found to chromosome 9 between markers rs6292345 and rs3698443 in a SNP analysis of 11 G3 *Tur/+* and 5 normal mice from a [C57BL/6J-*Tur/+* × C3HeB/FeJ] × C3HeB/FeJ backcross, narrowing the region of mutation to 11.8 Mb (Fig 2A). This linkage interval contained 77 known genes (http://genome.ucsc.edu), including the actin-based molecular motor *Myosin6* (*Myo6*) that is known to underlie forms of hereditary hearing loss in both humans and mice. We next performed whole-exome sequencing of DNA isolated from wild-type C3HeB/FeJ, wild-type C57BL/6J, *Tur/+* and *Tur/Tur* homozygous mice to identify whether there was a mutation in the *Myo6* gene in the *Tur* mutant. We focused analysis of whole-exome sequencing data on Chromosome 9 and identified a point mutation of A to T change in exon 8 of the *Myo6* gene (c.820A>T) only in *Tur* mutant mice and not in wild-type mice. No mutations were identified in other genes within the linkage interval region.

To confirm this mutation, we synthesized two independent primer sets flanking the mutation and performed PCR amplification using wild-type, *Tur/+* and *Tur/Tur* genomic DNA respectively. Direct sequencing of the amplified DNA fragments confirmed the presence of c.820A>T mutation in the *Tur* mice (Fig 2B) but not in the wild-type DNA samples tested. The c.820A>T mutation changes an asparagine to an isoleucine at position 200 (p.N200I) of the myosin VI protein. This asparagine residue is conserved in myosin VI proteins from human, mouse, pig, cat, chicken, sea urchin, *C*. *elegans* and *Drosophila* (Fig 2C).

### Degeneration of sensory epithelia in Tur mutant

**Fig 3:**
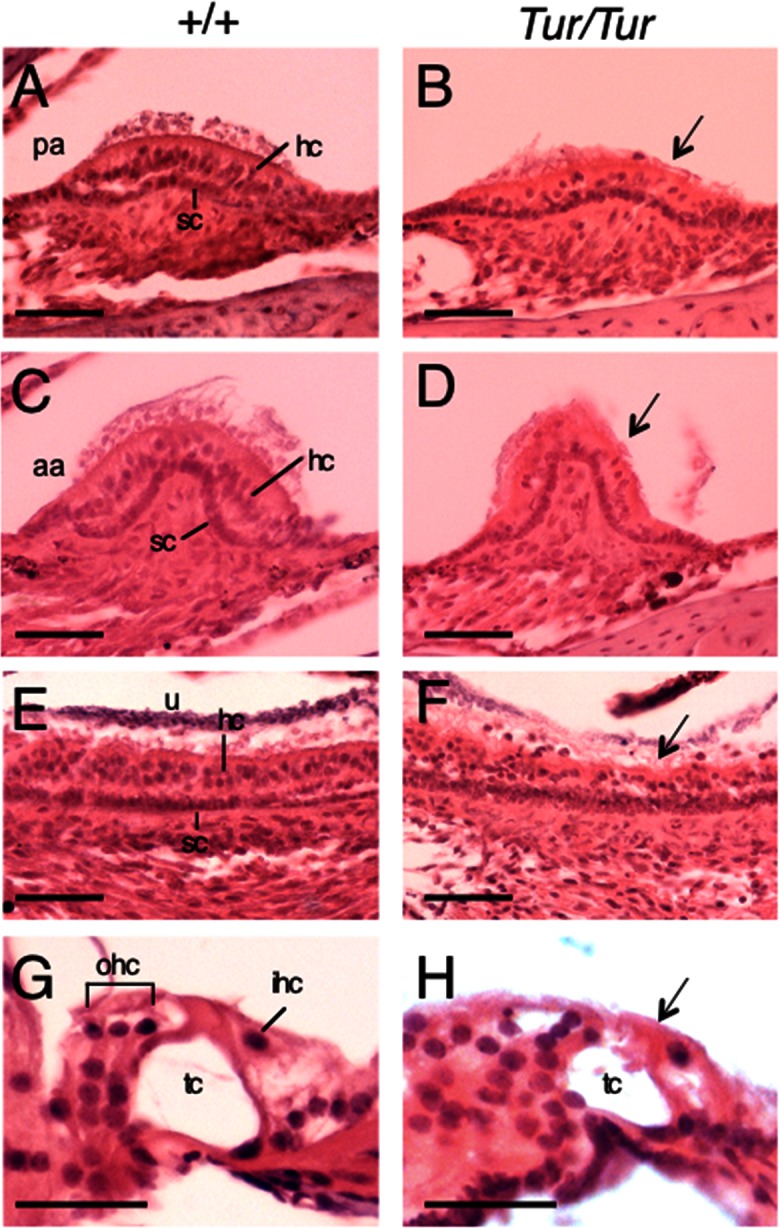
Degeneration of vestibular hair cells in *Tur/Tur* mutant. H&E staining of inner ear tissue at 4 weeks. (A-H) Posterior (A,B) and anterior (C,D) crista ampullaris, utricular macula (E,F) and Organ of Corti (G,H) in wild-type (A,C,E,G) and *Tur/Tur* mutant (B,D,F,H). Significant reduction of hair cells in the vestibular sensory organs (arrows in B,D,F) and collapse of the organ of Corti (arrow, H) are evident in the inner ear of *Tur/Tur* mice. Panels G,H are images showing the organ of Corti in the basal turn. Abb.: hc, hair cell; ihc, inner hair cell; ohc, outer hair cell; sc, supporting cell; tc, tunnel of Corti. Scale bar: A-F, 50 μm and G,H, 25 μm.

Histological analysis of four weeks-old wild-type and *Tur/Tur* mutant mice revealed a dramatic decrease in the density of hair cells in the cristae of all three semicircular canals responsible for detection of angular acceleration and in the maculae responsible for detection of linear acceleration (arrow, Fig 3B, 3D and 3F), resulting in much thinner sensory epithelia in the mutant compared to those in wild-type controls (Fig 3A, 3C and 3E). In the cochlea, collapse of the organ of Corti was noticeable at the basal turn with much smaller tunnel of Corti in the mutant (Fig 3H) compared to those in controls at this age (Fig 3G).

### Hair bundles of Tur mutant mice are severely disorganized

**Fig 4:**
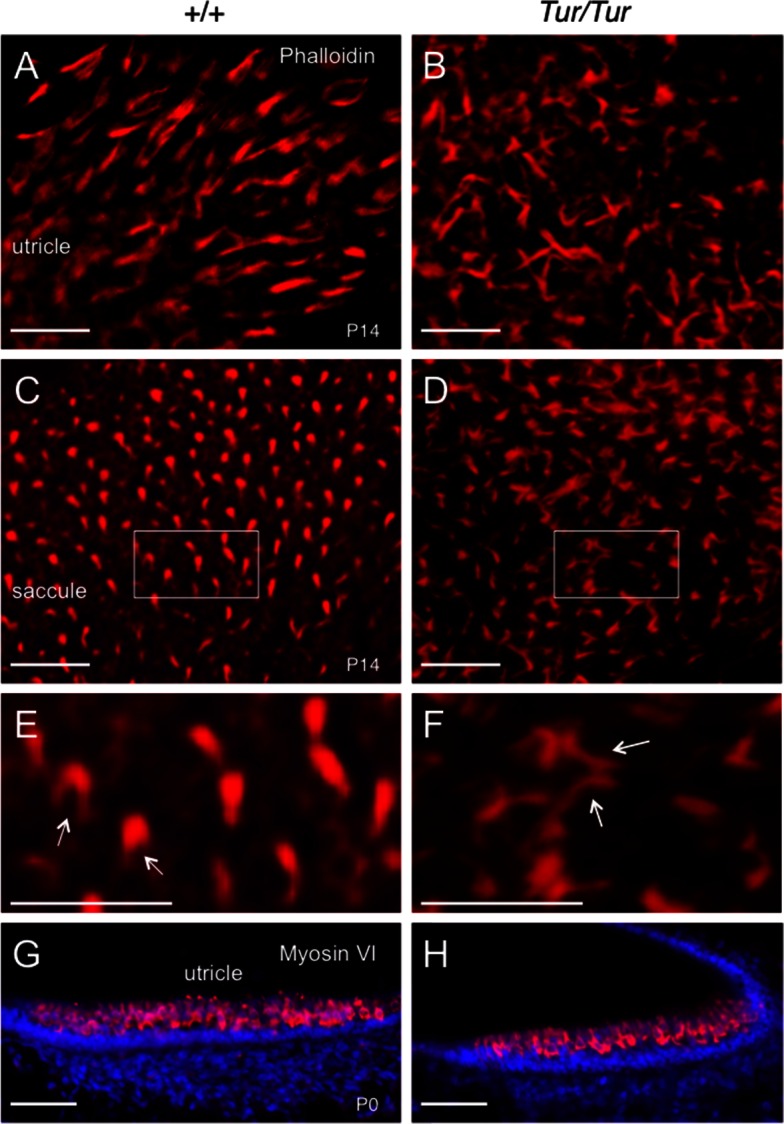
Disorganized vestibular hair bundles in *Tur/Tur* mutant. (A-F) Phalloidin staining showing stereocilia in maculae in the utricle and saccule at P14. Hair bundles of *Tur/Tur* mice are irregular with no pattern of organization. Arrows in F point to elongation of stereocilia in the mutant when compared to that in normal mice (arrows in E). Panel E,F are higher magnification of boxed area in C,D. (G,H) Anti-myosin VI immunostaining on utricle sections. Scale bars: E,F, 13 μm and all other panels, 20 μm.

Phalloidin staining was used to assess the hair bundles of the vestibular sensory epithelia (maculae of the utricle or saccule). At P14, the vestibular hair cells of *Tur/Tur* were visibly different from those of wild-type control mice as labeled by phalloidin (Fig 4A–4F), which specifically binds the filamentous actin of the stereocilia. The stereocilial hair bundles appeared to be elongated and misshapen with no pattern of organization in the mutant maculae (Fig 4B, 4D, 4F compare to 4A, 4C and 4E). We checked for Myosin VI protein expression and anti-Myosin VI immunostaining on sections revealed that the level of Myosin VI protein expression in the vestibular hair cells of *Tur/Tur* mutant at P0 (Fig 4H) was comparable to those of wild-type control (Fig 4G). No noticeable difference in the level of myosin VI protein expression was observed in all vestibular sensory epithelia between the wild-type and mutant mice (data not shown).

**Fig 5:**
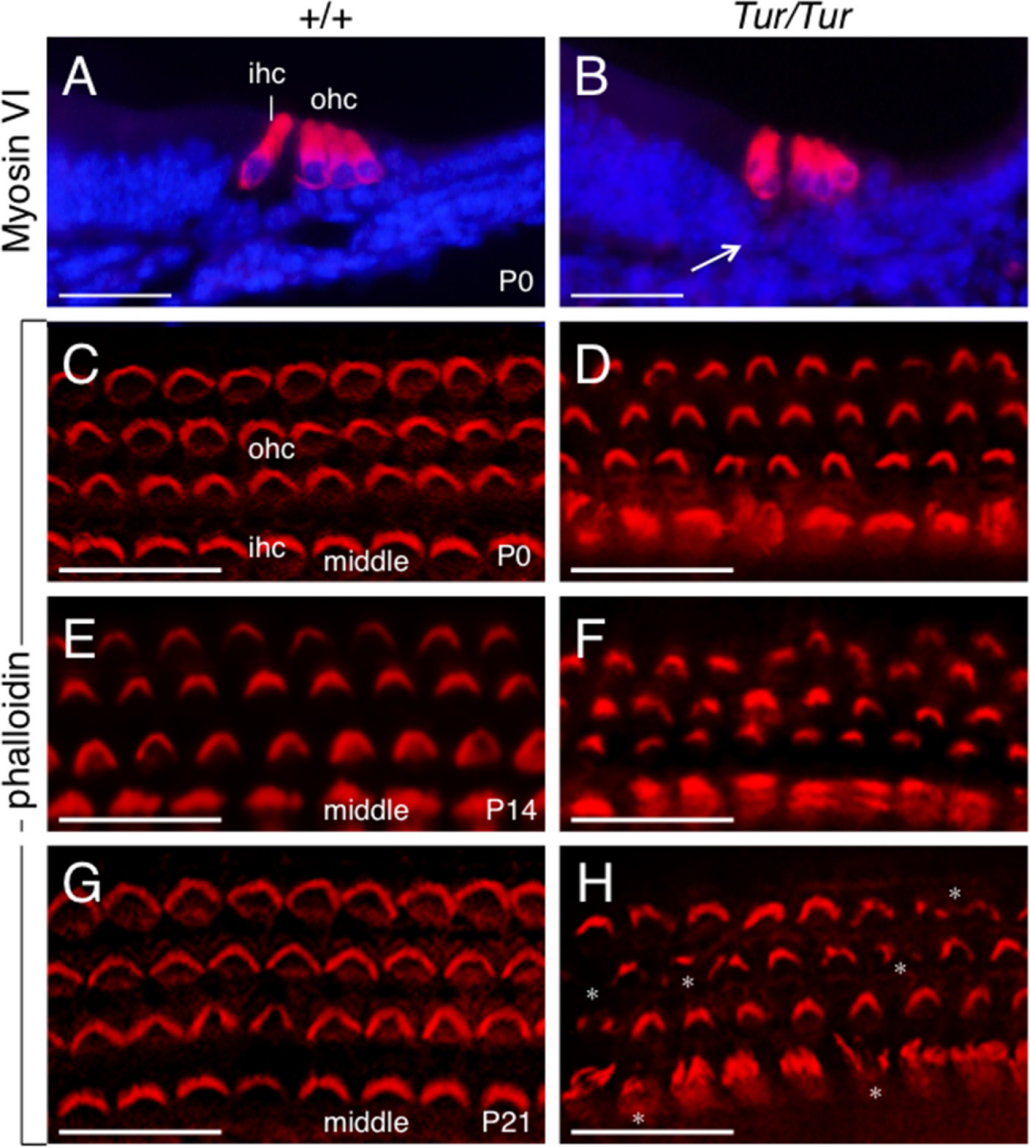
Progressive degeneration of hair bundles in *Tur/Tur* cochleae. (A,B) Immunofluorescence of anti-myosin VI on cochlear sections of wild-type (A) and *Tur/Tur* mutant at P0 (from middle turn). Arrow points to smaller tunnel of Corti in the mutant. (C-D) Phalloidin staining of whole-mount cochlear sensory epithelium (middle turn) from P0 (C,D), P14 (E,F) and P21 (G,H) with phalloidin (red). *Myo6*^*Tur/Tur*^ cochleae show irregular shapes of hair bundles compared to wild-type cochleae throughout all regions of the cochlea. Asterisks in H point to degeneration or elongation of stereocilial bundles in the mutant at P21. IHC, inner hair cell; OHC, outer hair cell. Scale bar: 25 μm.

In the cochlea, Myosin VI was expressed in hair cells of *Tur/Tur* mutant at P0 (Fig 5B) and its expression level appeared to be comparable to that in wild-type control (Fig 5A), even though the organ of Corti was smaller with smaller tunnel of Corti (arrow, Fig 5B). We next investigated cochlear hair bundle defects in the *Tur* mutant by performing staining for phalloidin and SEM analysis. Normal hair bundles have intrinsic structural polarity: stereocilia have graded heights, forming a crescent with the kinocilium centered next to the tallest stereocilia. At P0 in wild-type mice, all hair cells are well developed with their hair bundles aligned in a characteristic V-shaped pattern as labeled by phalloidin (Fig 5C). In the *Tur/Tur* mutant, overall the stereocilia maintain their V-shaped structure but hair bundles of both inner and outer hair cells in the mutant were disorganized and the normal V formation was disrupted and appeared smaller (Fig 5D). At P14, the stereocilial bundles in the mutant were misshapen and lacked the normal organization seen in wild-type hair cells (Fig 5E and 5F). By P21, structural disruption of the hair bundles was observed in the majority of cells in the mutant (split or degenerated) and elongated stereocilia were also apparent in the inner hair cells (asterisks, Fig 5H, compare to Fig 5G). These defects were observed along the length of the cochlea despite more advanced hair bundle degeneration in the basal and middle turns than in the apex.

**Fig 6:**
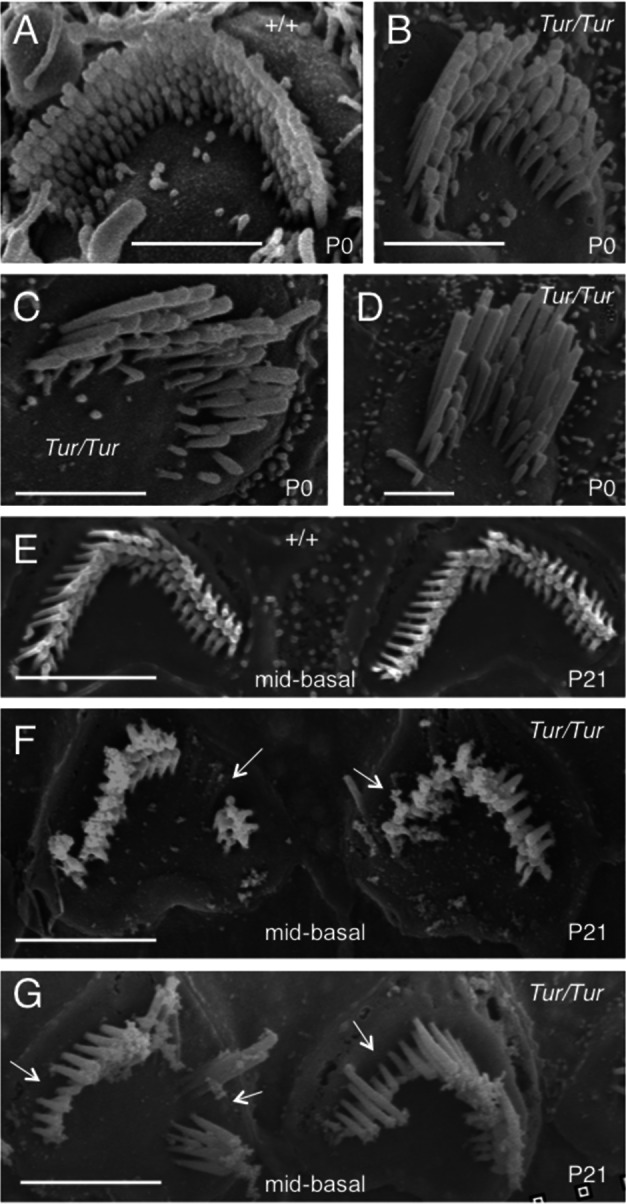
Disorganization and degeneration of hair bundles in *Tur/Tur* mutant cochleae at P0 and P21. SEM images showing stereocilial bundles of outer hair cells in the mid-basal turn in wild-type at P0 (A) and P21 (E), *Tur/Tur* at P0 (B-D) and P21 (F,G). *Myo6*^*Tur/Tur*^ cochleae show misshapen stereocilia, which appear to be smaller/narrower when compared to those in wild-type mice at P0. At P21, stereocilial degeneration is apparent in the majority of cells in the mid-basal turn (arrows). Scale bars: A-D, 2 μm; E,F, 3.0 μm; G, 2.5 μm.

**Fig 7:**
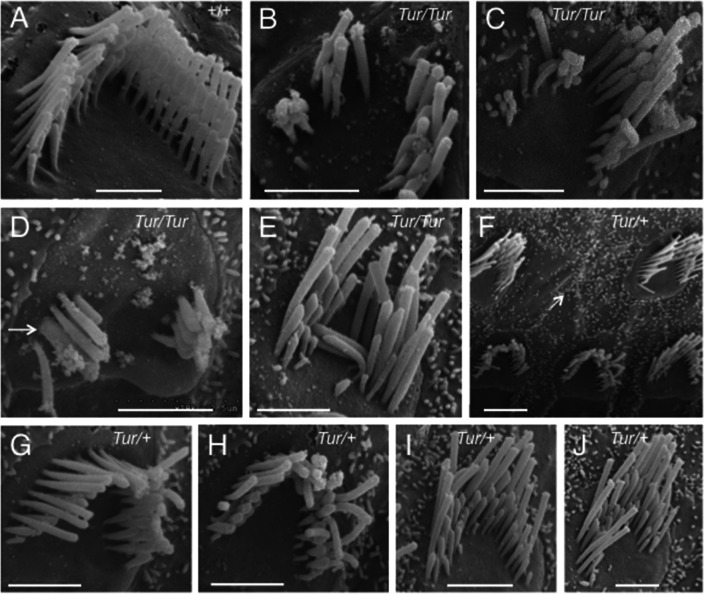
A variety of structural defects in *Tur* mutant cochlear hair bundles at P21. Higher magnifications of SEM images showing stereocilia bundles of outer hair cells in wild-type (A), *Tur/Tur* (B-E) and *Tur/+* (F-J) mice. *Myo6*^*Tur/Tur*^ cochleae show formation of multiple clumps of stereocilia within the same cell (split bundles) (B-D), stereocilial fusion bundles (arrow, D), folded stereocilia (E) or other abnormal shapes (generally deformed bundles) (C,E). In *Myo6*^*Tur/+*^ cochleae (F-J), the stereocilia also appear to be misshapen in the basal turn and loss of hair cells was occasionally observed (arrow in F). However, hair bundle split or degeneration was not apparent. Scale bars: A, 1 μm; B-E,G-J, 2 μm; F, 3 μm.

Close examination of the hair bundles in P0 and P21 wild-type mice with SEM analysis showed the characteristic staircase-like organization of stereocilia graded in height (Fig 6A and 6E). In contrast, in the *Tur/Tur* mutant at P0, the staircase-like arrangement of stereocilia of graded height was disrupted (Fig 6B and 6C) and the “V”-shaped stereocilia bundles were smaller than those in normal mice (Fig 6D). By P21, the characteristic staircase-like organization of stereocilia was unrecognizable and degeneration of hair bundles was obvious in the mid-basal turn (arrows, Fig 6F and 6G). Many hair bundles exhibited a variety of structural defects: formation of multiple clumps of stereocilia within the same cell (split bundles) (Fig 7B, 7C and 7D), stereocilial fusion (arrow, Fig 7D), folded bundles (Fig 7E) or other abnormal shapes (generally deformed bundles). In *Myo6*^*Tur/+*^ cochleae at P21, degeneration of hair cells was noticed in some areas in the basal turn (arrow, Fig 7F) and the stereocilial bundles were also disorganized (Fig 7G–7J). However, no apparent hair bundle split or degeneration was observed in the heterozygous mice at P21. No stereocilia branching was observed in the mutant.

## Discussion

In this study, we have identified a c.820A>T in *Myo6* responsible for inner ear defects such as headtossing, circling, and deafness displayed by our ENU mutant mouse strain *Turner*. Our genetic mapping identified the *Tur* mutation in the same chromosomal interval as *Myo6* and all *Tur* mutant mice identified by their abnormal behavior carried the c.820A>T mutation. This A>T transversion leads to a substitution of asparagine amino acid to isoleucine amino acid at position 200 (p.N200I), which is evolutionarily conserved in the motor domain with ATPase activity involved in actin binding and movement. Mutant protein levels appear to be normal as detected by the myosin VI antibody used in this study, which targets the C-terminal of the protein. N200 is in the beginning of the switch I loop (consensus sequence motif NxxSSR) [15] of myosin VI and given the general disorganization of hair bundles occurring in the *Tur* mutants, the p.N220I mutant protein is likely to hamper motor function of the myosin VI.

The motor domain at the N-terminus of the myosin superfamily is responsible for binding and moving along actin filaments. Thus far, myosin VI has been suggested to be involved in anchoring the apical hair cell membrane to the cuticular plate [16]. Myosin VI is also necessary for morphological and functional maturation of the inner hair cell ribbon synapses [17]. The abnormal hair bundle morphology in our *Turner* mice is consistent with the proposed role of *Myo6* in hair bundle morphogenesis. While slight disorganization of stereocilia is observed at P0, enhanced disorganization is apparent at P21. Hair cells have giant stereocilia and both inner and outer hair cells start to degenerate in *Tur/Tur* mice. Hearing loss and vestibular dysfunction with gradual sensory epithelial degeneration is likely related to an ongoing deterioration and fusion of stereocilia and finally hair cell death.

Consistent with the phenotype observed in our *Turner* mice, mislocalized kinocilium with hair bundles lacking the characteristic V shape, stereocilial fusion, and hair cell degeneration have been previously reported in other *Myo6* mutant mouse models with circling behavior and hearing loss: the spontaneous mutant Snell’s waltzer [2], and the ENU-induced mutants Tailchaser [18], twist [19] and charlie [12]. The Snell’s waltzer mouse (*Myo6*^*sv*^) or the charlie (*Myo6*^*chl*^) is predicted to represent a null allele as either mutation causes deletion of the coding region. The Tailchaser (*Myo6*^*Tlc*^) mutant carries an ENU-generated G>T transversion in exon 6, resulting in p.D179Y amino acid substitution in the motor domain of myosin VI [18]. Stereocilia branching was previously described in Tailchaser mice, but such defect was not observed in the *Tur* mutant. This phenotypic variation is likely caused by the two distinct mutations in the motor domain of the myosin VI. Indeed, different mutations in *MYO6* are known to result in differences in hearing loss, as seen in previously described DFNA22 families i.e. differences in age of onset (early to late onset), progression and severity (moderate to profound) [5, 7, 20, 21]. It has been suggested that missense mutations that affect the motor domain of myosin VI lead to a more severe hearing impairment and an earlier onset. While *Turner* and *Tailchaser* carry different mutations in the motor domain, the phenotypic variation between these two mutations in the motor domain provides novel insight into different aspects of protein function.
